# The sterile insect technique for the management of the spotted wing drosophila, *Drosophila suzukii*: Establishing the optimum irradiation dose

**DOI:** 10.1371/journal.pone.0180821

**Published:** 2017-09-28

**Authors:** Geneviève Lanouette, Jacques Brodeur, François Fournier, Véronique Martel, Marc Vreysen, Carlos Cáceres, Annabelle Firlej

**Affiliations:** 1 Institut de recherche et de développement en agroenvironnement, Saint-Bruno-de-Montarville, Québec, Canada; 2 Institut de recherche en biologie végétale, Université de Montréal, Montréal, Québec, Canada; 3 Collège Montmorency, Laval, Québec, Canada; 4 Centre de foresterie des Laurentides, Ressources naturelles Canada, Québec, Québec, Canada; 5 Insect Pest Control Laboratory, Joint FAO/IAEA Division of Nuclear Techniques in Food and Agriculture, Seibersdorf, Austria; Institute of Plant Physiology and Ecology Shanghai Institutes for Biological Sciences, CHINA

## Abstract

The spotted wing drosophila *Drosophila suzukii* Matsumura (Diptera: Drosophilidae), a pest of berries stone fruits, invaded North America and Europe in 2008. Current control methods rely mainly on insecticides. The sterile insect technique (SIT) has potential as an additional control tactic for the integrated management of *D*. *suzukii*. As a step towards the development of the SIT, this study aimed at finding the optimum irradiation dose to sterilize *D*. *suzukii* under controlled laboratory conditions. Four-day-old *D*. *suzukii* pupae were irradiated 12 to 24 hours prior to adult emergence in a ^60^Co Gamma Cell 220 and in a ^137^Cs Gamma Cell 3000 with doses of 30, 50, 70, 80, 90, 100 or 120 Gy. Emergence rate (88.1%), percent of deformed flies (4.0%) and survival curves were not affected by the tested irradiation doses. However, some reproductive parameters of the flies were affected by irradiation. Females irradiated with a dose of 50 Gy or more had almost no fecundity. When non-irradiated females were mated with irradiated males, egg hatch decreased exponentially with irradiation dose from 82.6% for the untreated control males to 4.0% for males irradiated with 120 Gy. Mortality of F1 individuals from the irradiated treatment also occurred during larval and pupal stages, with an egg to adult survival of 0.2%. However, descendants produced by the irradiated generation were fertile. These results are an encouraging first experimental step towards the development of the SIT for the management of *D*. *suzukii* populations.

## Introduction

*Drosophila suzukii* Matsumura (Diptera: Drosophilidae) is an invasive pest of berries and stone fruits. It is a native from Southeast Asia and was first reported in North America (California) and Europe (Spain) in 2008 [1, 2]. By 2010, the fly was present in nine states in the USA and four Canadian provinces [1] and in 2013 had reached South America [3]. Unlike other *Drosophila* species that thrive on decaying fruits, *D*. *suzukii* has a serrated ovipositor that allows females to lay eggs in ripening and marketable fruits [4]. Not only will the developing larvae damage the fruit, but the puncture made by the ovipositor is an entry point for pathogenic fungi and bacteria [5]. Initially a pest of cherries in Asia, *D*. *suzukii* has expanded its host range to more than 15 commercial plant species including raspberries, blueberries, strawberries and grapes within invaded regions where severe damaged are observed [4]. Between 2009 and 2014, California alone experienced revenue losses of $36.4 million and $3.4 million in conventional and organic production, respectively [6]. To protect crops from *D*. *suzukii* infestation, growers mostly have been using chemical insecticides [7–10], requiring an average of four to six applications per growing season [6, 11]. Insecticide use leads to environmental pollution, public health issues, pest resistance and mortality of natural enemies [12]. Alternative methods are urgently needed to reduce our dependence on insecticides for the sustainable management of *D*. *suzukii*.

Alternative methods to insecticides, effective or under evaluation, include mass-trapping of adult flies using attractants [13, 14], protecting crops with exclusion nets [15], cultivating crops inside closed tunnels [16], spraying crops with natural repellents [17, 18], harvesting more frequently [4] and postharvest refrigeration, fumigation or irradiation [19–21]. Other avenues include the use of native and/or exotic parasitoids [22–24], generalist predators [25] or entomopathogenic fungi [26]. One alternative approach that remains unexplored is the sterile insect technique (SIT).

The SIT consists of mass-rearing the insect pest in specialized facilities, exposing pupae or adults to ionizing radiation to induce reproductive sterility and releasing them in the target area. The released sterile males will mate with virgin wild females and, as a result, the females will lay unfertile eggs. A sustained release of sterile males in a target area aims at reducing the pest population over time below an acceptable economic threshold, or even reaching local eradication in certain ecological settings [27]. The sterilization of males and females is achieved by ionizing radiation, mainly using gamma or X rays which have high levels of energy and penetration power [28, 29]. The irradiation triggers lethal mutations in the sperm and, following insemination of the oocyte, the death of the developing embryo [30].

The SIT has been used effectively against many crop and livestock pests and disease vectors. Following the successful eradication of the New World screwworm *Cochliomyia hominivorax* Coquerel (Diptera: Calliphoridae) from the USA, Mexico, Central America and Panama [31], the technique has been increasingly used worldwide for the management of several Tephritidae fruit flies, tsetse flies and Lepidoptera [32]. Its success depends on the knowledge of the pest biology, the competitiveness of the irradiated males and the sustained and area-wide release of sterile insects [33, 34].

A crucial and initial step in the development of an SIT program is to determine the optimal irradiation dose for the targeted pest species. The optimal dose should sterilize individuals without impairing critical traits of their biology such as their ability to mate. Males exposed to high irradiation doses are potentially less likely to compete and mate with wild females than non-irradiated males. A dose inducing 100% sterility is rarely used as it usually incurs excessive somatic damage to the insects [30, 35]. For example, Toledo et al. [36] found that an irradiation dose of 40 Gy induced an average of 99.5% sterility in male *Anastrepha obliqua* Macquart (Diptera: Tephritidae) and that these irradiated males were twice as effective in increasing the amount of sterile eggs in the population than fully sterile males irradiated with 80 Gy. For an optimal use of the SIT, irradiation should cause high sterility without affecting emergence rate, rate of deformed males, adult longevity, and the fecundity of healthy females mated with irradiated males.

The biological quality of irradiated males is assessed using various parameters, e.g. percentage adult emergence, percentage deformed insects, flight ability, longevity with or without access to food, fecundity of inseminated females (number of eggs produced), fertility (percentage of hatching), presence or absence of sperm transfer, male mating competitiveness, and overall capacity to reduce pest populations under semi-field or field conditions [37–41].

This study is part of a large research program that aims to determine the feasibility of using the SIT as an additional control tactic for the integrated pest management of *D*. *suzukii* populations in fruit crops. The present objective is to quantify the effects of different gamma irradiation doses applied to *D*. *suzukii* pupae on several biological attributes of irradiated individuals and their descendant (emergence, deformed males, longevity, fecundity, fertility of parent and descendant flies).

## Material and methods

### Insect colonies and rearing method

A *D*. *suzukii* colony was established from individuals collected in a vineyard near San Michele all Adige, Trentino, Italy. The colony had been in culture for one year before being sent to the Insect Pest Control Laboratory (IPCL) of the Joint Food and Agriculture Organization of the United Nation (FAO)/International Atomic Energy Agency (IAEA) Division of the Nuclear Techniques in Food and Agriculture in Seibersdorf, Austria. A first set of laboratory experiments (fertility and fecundity tests) were carried out at the IPCL and the colony was thereafter sent to the Institut de Recherche et de Développement en Agroenvironnement (IRDA), St-Bruno-de-Montarville, Canada, where a second set of experiments was carried out (fecundity, emergence, deformity, longevity, F1’s survival and F1’s fertility tests). The colony was kept at 23 ± 1°C, 50 ± 10% HR, and under a 16:8 L:D photoperiod. Two types of larval diet were used: a carrot powder diet developed for the Mediterranean fruit fly *Ceratitis capitata* Wiedemann (Diptera: Tephritidae) [42] and a fresh banana diet developed for *D*. *suzukii* [22]. The carrot diet was used to produce large and uniformly sized pupae to be irradiated and for fecundity tests. The banana diet was used for the fertility, F1 survival and F1 fertility experiments because it provides higher egg to pupa survival (survival of 22.1% in previous unpublished test using carrot diet showed in S7 Table vs. survival of 72.1% in banana diet in present study using banana diet). When used as oviposition site, the carrot diet was spread inside a 1L rectangular container, covered with thin slices of fresh banana and put inside a rearing cage for 2 to 3 days to attract adult females. Thereafter, the diet was removed from the cage, covered with a mesh and placed under rearing conditions for larval development. Pupal extraction was done on days 6, 7 and 8 after oviposition using soft clips. Pupae were briefly washed in water and deposited on a wet makeup cotton pad. Following emergence, adults had access to water and a diet composed of white sugar and brewer’s yeast (3:1). Adults were reared in 29 X 29 X 29 cm plexiglass cages ventilated with muslin netting.

### Irradiation

Four day-old *D*. *suzukii* pupae (12 to 24 h prior to adult emergence) were irradiated in a ^60^Co Gamma Cell 220 (MDS Nordion, Canada) for experiments conducted at the IPCL and in a ^137^Cs Gamma Cell 3000 (Best Theratronics, Canada) at the Centre de Recherche du Centre Hospitalier de l’Université de Montréal (CRCHUM) for experiments in Canada. Gamma rays emitted by ^60^Co or ^137^Cs are similar (C. Cáceres, personal communication), but the two radiation sources differed in their dose rate, which is known to have no effect on emergence, longevity and flight ability of tephritid fruit flies such as *Dacus cucumis* French [43] and *Bactrocera tryoni* Froggatt [44]. Experiments conducted in Austria and Canada can therefore be merged. Pupae for irradiation and control pupae were transported in a thermal bag to the irradiation center and were thus exposed to the same temperature and humidity conditions. The irradiation doses used in all experiments were 30, 50, 70, 80, 90, 100 or 120 Gy, whereas control pupae were not irradiated.

### Adult emergence and deformed flies

A first objective was to correlate irradiation dose applied to pupae with percentage of adult emergence and deformed flies. We first developed a method to allow sexing of the flies and to assure that all individuals were virgin before being transferred to rearing cages following irradiation. A few hours following irradiation, *D*. *suzukii* pupae were placed individually in the wells of an ELISA plate identified with irradiation dose and date and covered with Parafilm®. On the following day, plates were observed every few hours from morning to late afternoon for adult emergence. From visual observation, without using anesthesia, each fly was identified through the plastic plate as male or female, and as being healthy or deformed. Sex was assessed through the presence/absence of an ovipositor at the end of the abdomen. Deformed flies included individuals that partially emerged from the pupal case, and individuals whose wings did not fully deploy following emergence. Healthy flies were removed from the plate with an insect aspirator shortly after emergence and used in experiments. The plates were observed for an additional day for late emergence, and the remaining unemerged pupae were classified as dead individuals. A total of 8,958 pupae were examined (~3000 pupae from the control and ~800 pupae for each experimental doses), allocated in 11 replicates (irradiation dates) at CRCHUM. To test for an irradiation dose effect on adult emergence and deformity, two binomial generalized linear models were used in R 3.3.2 [45].

### Longevity

We tested the effect of irradiation on the average lifespan of virgin adults. Upon emergence and sexing, *D*. *suzukii* adults were placed in groups of ten flies of the same sex in a 15 X 15 X 15 cm plastic cage with access to water and adult diet (white sugar and brewer’s yeast). Mortality was recorded each day at 9 AM until all flies had died. Two cages of ten virgin females and two cages of ten virgin males were set up for each dose and the experiment was repeated four times (n = eight cages, for a total of 80 individuals, per sex and per dose). Longevity per dose was analyzed using Kaplan-Meir survival analyses and, for each sex, survival curves were compared using Mantel-Cox log-rank tests using R 3.3.2 [45].

### Fecundity

The effect of irradiation dose on fecundity was assessed for (1) ten non-irradiated females mated with ten irradiated males, (2) ten irradiated females mated with ten non-irradiated males and (3) ten non-irradiated virgin females. After emergence and sexing, *D*. *suzukii* adults were assigned to one of the three treatments. Flies were placed in cages similar to the ones used for longevity test with access to water, adult diet, and an egg-laying site consisting of a Petri dish (4 cm diameter) filled with carrot diet covered with slices of fresh banana. The egg-laying site was renewed three times a week. Following the pre-oviposition period, oviposition was monitored for seven days. Following oviposition, egg-laying sites were immediately observed under a stereomicroscope. *Drosophila suzukii* eggs have two visible white filaments near the anterior end coming out above the surface of the diet and allowing the embryo to breathe, which enable precise egg counting [46]. For analysis, the numbers of eggs per cage were pooled for the seven-day period.

For each male-female combination, two cages containing ten couples were set up per treatment. The experiment was replicated four times (n = eight cages per combination per dose for mated flies and n = 13 cages for virgin females). This experiment was conducted at both the IPCL and the IRDA, but virgin females were tested only at the IPCL. Data were analyzed separately for irradiated males and females. For irradiated males, a mixed model was used to examine the effect of irradiation dose on fecundity (total number of eggs laid in seven days per cage containing ten couples), adding the irradiation date as a random variable to consider possible differences between irradiation sites. For irradiated females, a mixed model with Poisson distribution and Dual Quasi-Newton test for over dispersion of data were used to examine the effect of irradiation dose on fecundity. Analysis was done using R 3.3.2 [45].

### Fertility

The effect of irradiation dose on male fertility was examined by assessing egg hatch when non-irradiated females were mated with irradiated males. After emergence and sexing, ten irradiated adult males were place together with ten non-irradiated females in a cage with access to water, adult diet, and a banana slice. Flies were left together for five days for sexual maturation and mating. Thereafter, with both male and female flies present in the cages, the banana slice was changed twice a day for three consecutive days to sample the eggs. Each day, one of the banana slices was checked under a stereomicroscope (the first one was from 8 AM to 4 PM, the second and third were from 4 PM to 8 AM), and all eggs were carefully taken out with forceps and put on a black filter paper placed on a wet sponge. Eggs were incubated at rearing conditions for 48 h, after which, egg hatch was scored under a stereomicroscope. An average of 150 ± 62 eggs was harvested per cage over three days. Percent hatch was calculated for each cage and used as a measure of male fertility. Two cages containing ten couples were set up per treatment, except for the first replicate when three cages were used per treatment. The experiment was replicated five times (n = eleven cages per treatment). The data were analyzed using a non-linear method (NLIN) describing the decreasing exponential function using SAS 9.4 [47].

### F1 survival

Survival of F1 larvae, i.e. descendants of parents of which the male had been exposed to irradiation, was assessed. In contrast to the previous fertility experiment, we used the banana diet because it provided higher survival of *D*. *suzukii* larvae. Following adult emergence and sexing, ten irradiated males were placed together with ten non-irradiated females in a cage with access to water, adult diet and 25 ml of banana diet poured into a 4 oz Solo cup without cover as oviposition site. The oviposition site was changed three times a week for two weeks. The eggs on the oviposition site were taken out of the cage and counted under a stereomicroscope. A muslin-covered top was put on the Solo cup that was kept under rearing conditions for eight days, and humidified when necessary. Then, the patch was re-examined for counting the number of pupae formed on top of the diet. The pupae were carefully taken out with forceps and put on a wet makeup cotton pad in a new clean Solo cup of the same size with muslin-covered top. The cotton pad was re-humidified when necessary until adult emergence. Adults were thereafter sexed and counted. The number of eggs, pupae and adults of each sex produced in two weeks were determined for each cage. Two cages containing ten couples were set up per treatment. The experiment was repeated four times (n = eight cages per treatment) and the data were analyzed using a non-linear method (NLIN) describing the decreasing exponential function using SAS 9.4 [47].

### F1 fertility

We next assessed the fertility of F1 descendants (produced in the previous experiment), i.e. descendant flies from crossings of partially sterile irradiated males with non-irradiated females (F0). Inherited sterility has been observed in several insect species, particularly in Lepidoptera [48]. Only descendants produced from pairs where the males had been irradiated at doses of 70 Gy and higher were tested. Upon emergence, each descendant was put individually in a 1 oz Solo cup with a muslin-covered top, 5 ml of banana diet and two non-irradiated adults of the opposite sex. Those two non-irradiated adults were replaced if they died before the end of the test. The diet was changed three times during a ten-day period. The diet was humidified and kept for eight days to determine the total number of pupae produced by each descendant. If the descendant was a male, the number of pupae produced was divided by two since he had been coupled with two females. Since the F0 couples with males irradiated at doses of 70 Gy or more produced few descendants, the number of individuals for this experiment was low, i.e. from two to eleven descendants per dose. Only average and standard deviation of the number of descendants produced per female by each F1 individuals were calculated using R 3.3.2 [45].

## Results

### Adult emergence and deformed flies

**Fig 1:**
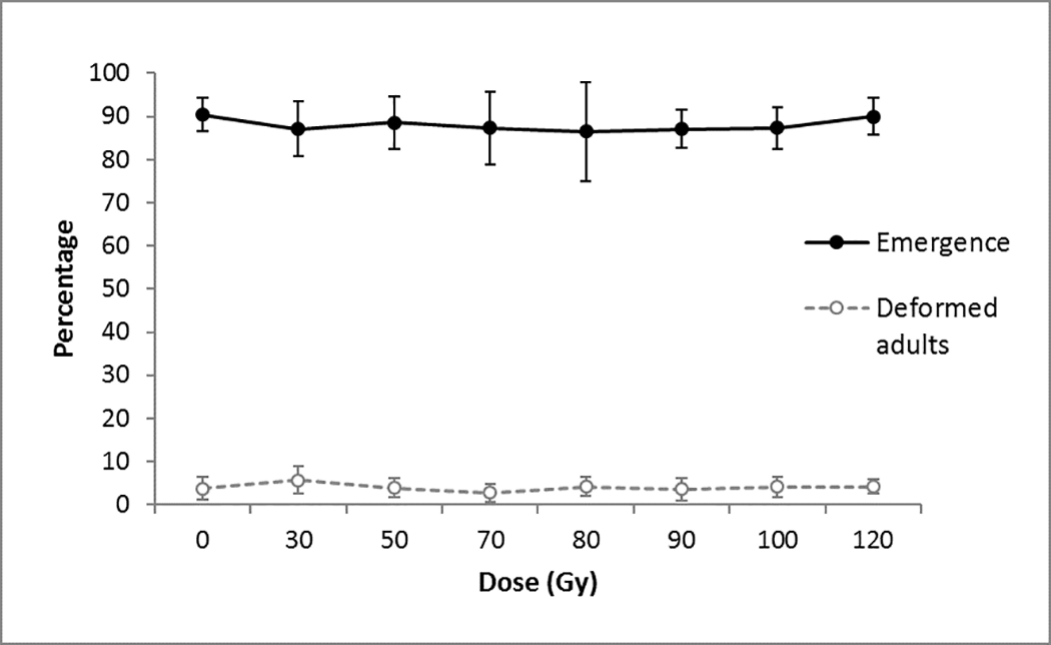
Effect of irradiation dose on percent *D*. *suzukii* adult emergence and percentage deformed adults. Significances were measured with binomial generalized linear models (P> 0.05).

For each dose, gamma ray irradiation of four-day-old *D*. *suzukii* pupae did not have a significant effect on adult emergence (Binomial generalized linear model; F = 0.2031, d.f. = 7, P = 0.663) and percentage deformed adults (Binomial generalized linear model; F = 0.3031, d.f. = 7, P = 0.580). For each treatment, percentage emergence was high (88.1 ± 6.5%), and percentage deformed flies was low (4.0 ± 2.4%) (Fig 1).

### Longevity

**Fig 2:**
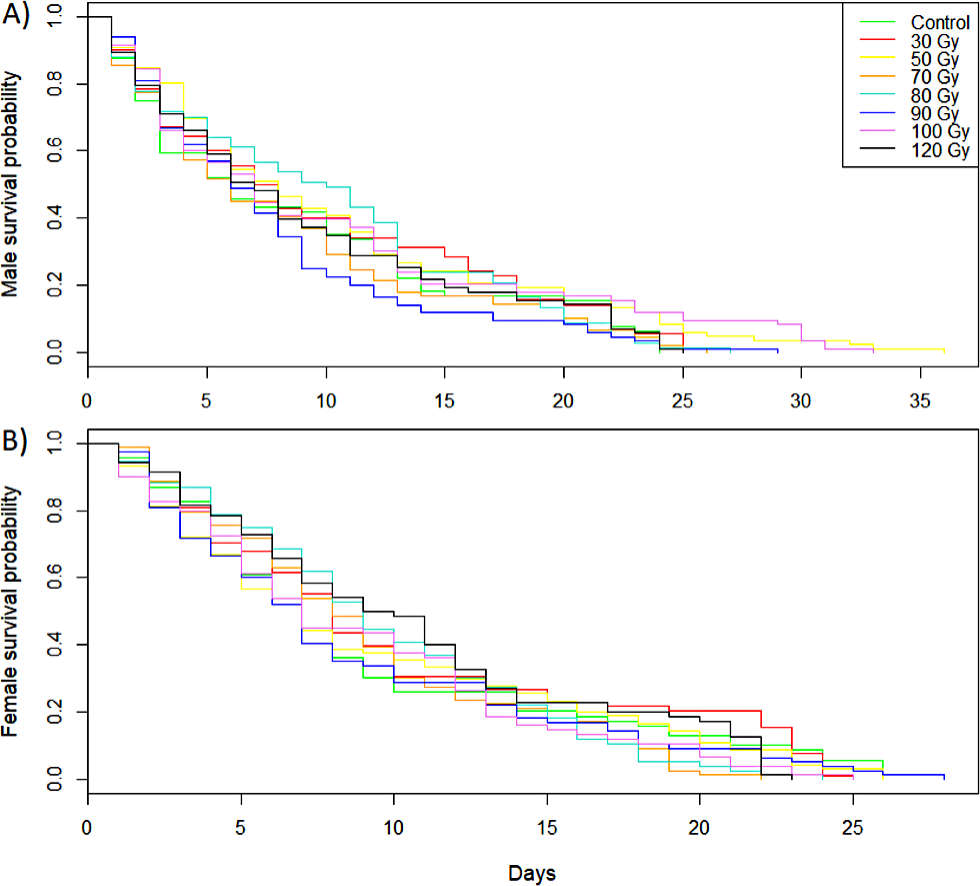
**Effect of irradiation dose on longevity of *D*. *suzukii* (A) males and (B) females when flies had access to food and water.** Significance were measured with Mantel-Cox log-rank test (P>0.05).

For both males and females, irradiation did not have a significant effect on longevity (Mantel-Cox log-rank; X^2^ = 13.5, d.f. = 7, P = 0.062 for males; X^2^ = 5.2, d.f. = 7, P = 0.635 for females) (Fig 2). Males survived from 1 to 36 days while females survived from 1 to 28 days.

### Fecundity

**Fig 3:**
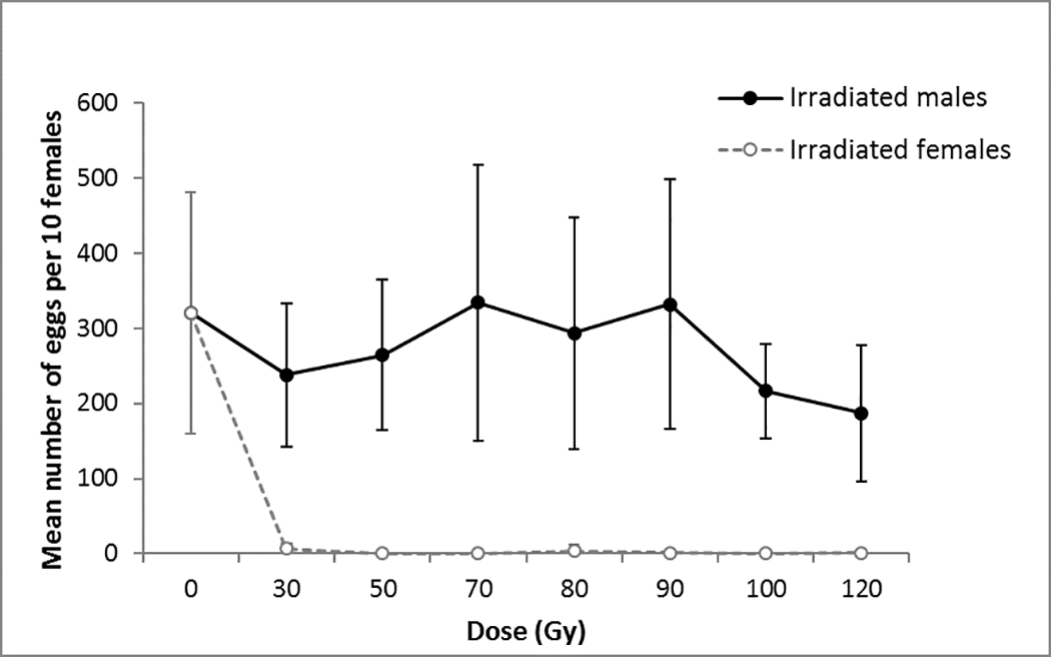
Effect of irradiation dose on *D*. *suzukii* fecundity (number of eggs oviposited in one week per ten couples) when either males or females were irradiated. Significance was measured with a Linear mixed model for irradiated males (P>0.05) and a Poisson generalized linear model for irradiated females (P<0.0001).

On average, 320.4 ± 160.0 eggs were laid per week in control cages, while 275.8 ± 140.9 eggs were laid in cages containing ten irradiated males at any dose and ten non-irradiated females. The number of eggs sampled in one week did not differ significantly with irradiation dose given to the males (control consisted of non-irradiated males and non-irradiated females) (Linear mixed model, F = 0.2290, d.f. = 7, P = 0.634). In cages containing ten non-irradiated males and ten irradiated females, all irradiation doses drastically reduced the fecundity of *D*. *suzukii* females (Poisson generalized linear model, F = 53.52, d.f. = 7, P<0.0001). Females irradiated with 30 Gy laid 6.3 ± 2.0 eggs and less than one egg per cage was found for all other doses, except with 80 Gy where females had a fecundity of 3.6 ± 1.3 eggs per cage. No difference was found in fecundity of females irradiated with doses of 50 Gy and more (Multiple t-tests, P>0.05). Virgin females also laid sterile eggs, with an average of 34 ± 40 eggs per week per ten females (Fig 3).

### Fertility

**Fig 4:**
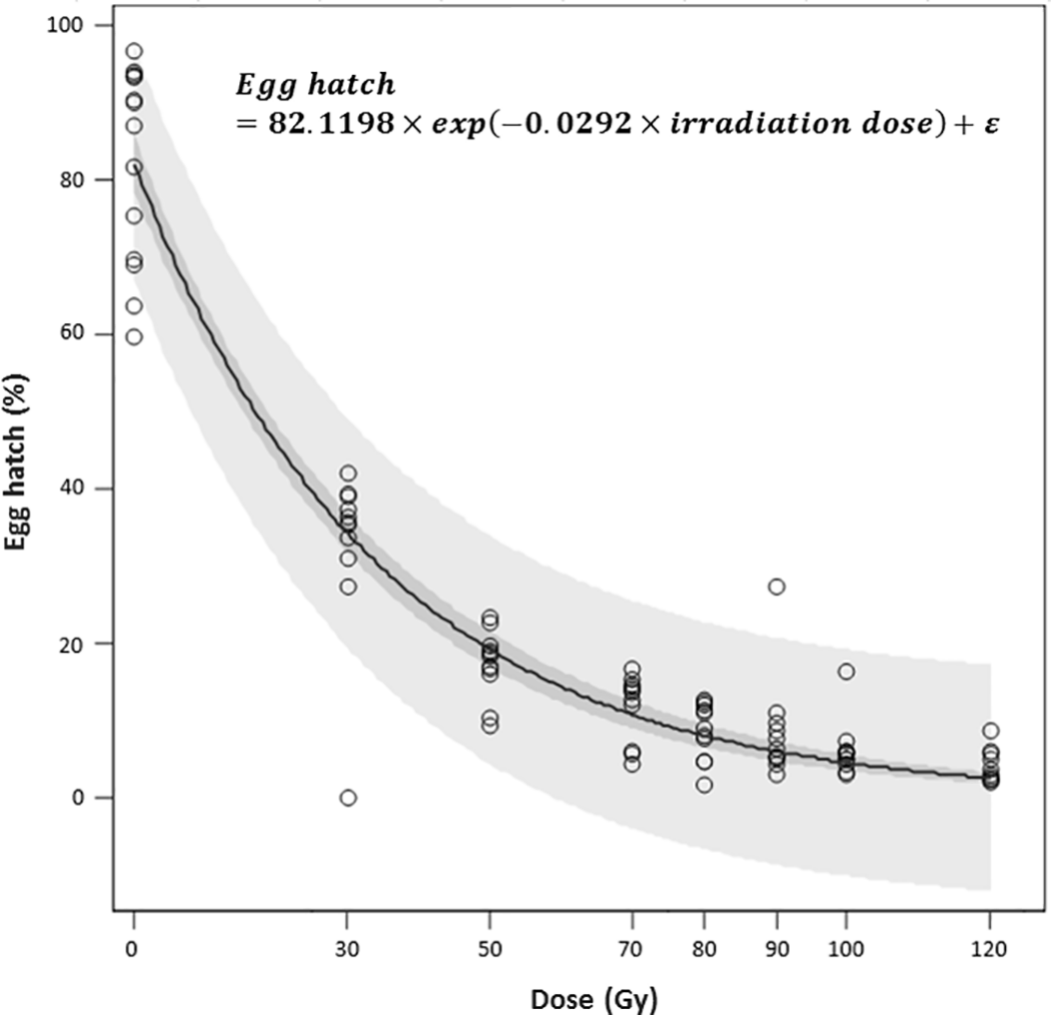
Effect of irradiation dose on egg hatch when non-irradiated *D*. *suzukii* females were mated with irradiated males. Dark and pale shaded areas represents 95% confidence limits and 95% prediction limits, respectively.

The hatchability of eggs laid by non-irradiated *D*. *suzukii* females mated with irradiated males decreased exponentially with irradiation dose (Regression, pseudo-R^2^ = 0.93); i.e. from 82.6% in the untreated control cages to 4.0% in cages with males irradiated with 120 Gy (Fig 4).

### F1 survival

**Fig 5:**
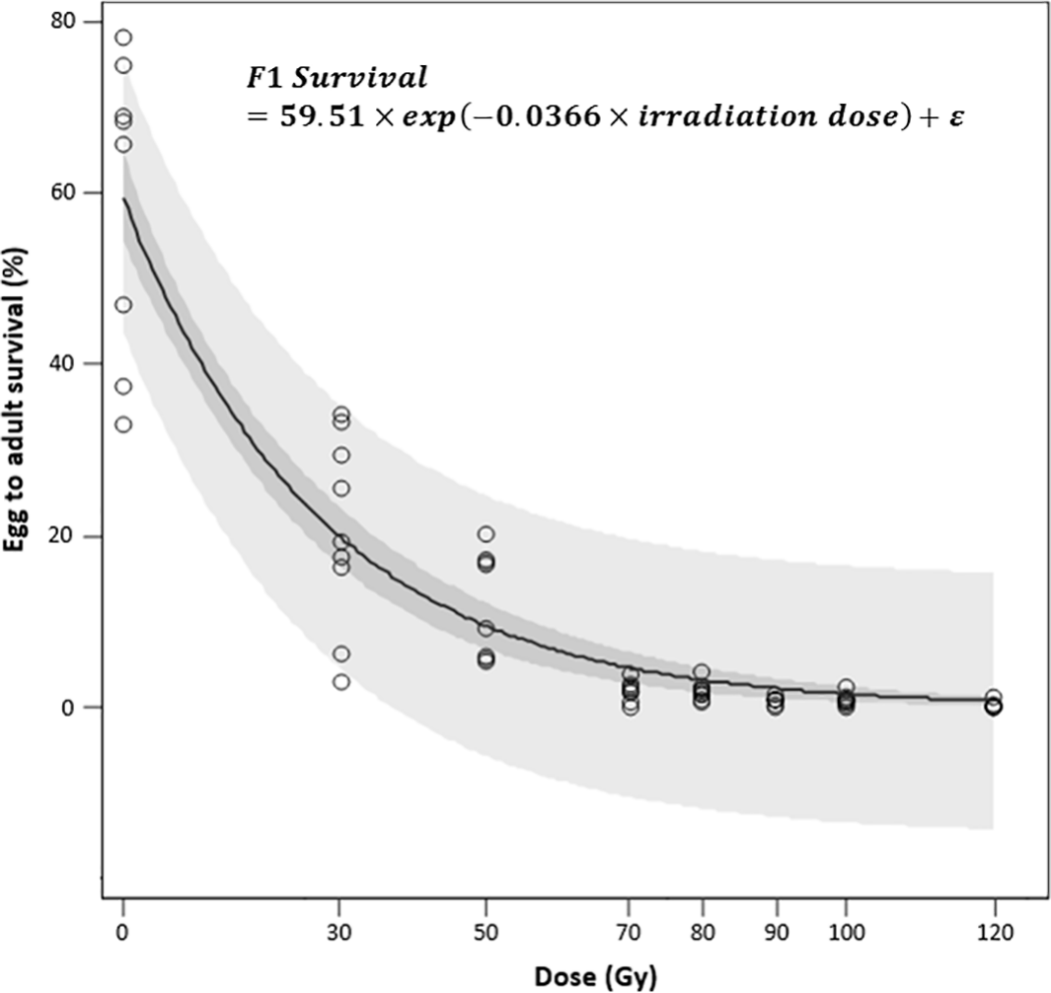
Effect of irradiation dose on survival to adult stage of eggs laid by non-irradiated *D*. *suzukii* females when mated with irradiated males. Dark and pale shaded areas represents 95% confidence limits and 95% prediction limits, respectively.

Survival from egg to adult of the F1 generation decreased with irradiation dose following an exponential regression curve (pseudo-R^2^ = 0.86), i.e. from 59.2% in the control cages to 0.2% in cages with males irradiated with 120 Gy (Fig 5). The difference between the egg hatch curve and the survival from egg to adult results from natural mortality at the larval and pupal stages.

### F1 fertility

Too few data were obtained on the fertility of the descendants to be statistically analyzed. Nevertheless, results presented in Table 1 suggest that individuals from the F1 generation that reached the adult stage were fertile, regardless of the irradiation dose of the F0, except for the only F1 female obtained from the 120 Gy irradiated F0.

**Table 1:** Effect of gamma irradiation dose applied to *D*. *suzukii* males on the fertility of males and females of the F1 generation.

Irradiation dose (Gy)	Descendants produced by females F1 (pupae ± SD)	*n*	Descendants produced by males F1 (pupae ± SD)	*n*
70	34.5 ± 28.5	4	46.5 ± 46.3	3
80	22.8 ± 21.0	5	46.0 ± 32.5	6
90	5.3 ± 5.51	3	9.7 ± 9.9	4
100	21.7 ± 28.2	3	64.9 ± 65.9	4
120	0	1	57.0	1

*n* = number of F1 descendants.

## Discussion

The objective of the present study was to assess the effects of gamma irradiation on several biological attributes of *D*. *suzukii*. The selection of the optimal irradiation dose for use in an SIT program is a crucial step that requires careful evaluation under laboratory conditions. The optimal dose should balance high levels of insect sterility with minimal impact on their overall biological quality [35]. A small residual fertility can be accepted in situations where the competitiveness of irradiated males is preserved [29, 36]. The optimal dose is thus a trade-off between complete sterility and alterations induced in the somatic cells by irradiation.

Gamma irradiation did not cause apparent morphological damage to males and females *D*. *suzukii*, even at the highest dose tested (120 Gy). Provided that these results are confirmed by further evaluation of the competitiveness of irradiated males under laboratory and field conditions, this suggests that released irradiated males would be as successful as wild males to find and mate with wild females. In addition, sterile insect production facilities will not suffer losses from non-emerging pupae or deformed adults. Such a pattern has been observed in other insect species: irradiation up to 100 Gy did not impair *Anopheles arabiensis* Patton (Diptera: Culicidae) emergence and longevity [39]; irradiation with 170 Gy had no effect on emergence and longevity, with or without access to food, of the American serpentine leafminer *Liriomyza trifolii* Burgess (Diptera: Agromyzidae) [40]. On the other hand, Amoako-Atta et al. [38] showed that irradiation of the almond moth *Cadra cautella* Walker (Lepidoptera: Pyralidae) with 100 to 300 Gy as late stage pupae had no effect on adult emergence but percentage deformed adults increased from 2 to 10%, while irradiation of younger pupae induced high mortality and a high percentage of deformed adults.

*Drosophila suzukii* males and females cannot be differentiated at the pupal stage. Since both sexes will have to be released in the field, it is crucial to ensure complete female sterility following irradiation. Females irradiated with doses of 50 Gy and higher were sterile, indicating that they are more sensitive to gamma irradiation than males. Irradiation with 50 Gy did not fully sterilized males; when mated with a non-irradiated female, egg hatching and survival from egg to adult were reduced to 17.3% and 12.2%, respectively. This higher radio-sensitivity of females facilitates the development of an SIT program as it allows the selection of the optimal dose for males. Difference in radio-sensitivity between sexes is a common finding in many insects like the beet leafhooper *Circulifer tenellus* Baker (Hemiptera: Cicadellidae) [49], the almond moth [50], the Indian mealmoth *Plodia interpunctella* Hübner (Lepidoptera: Pyralidae) [51] and the South American fruit fly *Anastrepha fraterculus* Wiedemann (Diptera: Tephritidae) [37].

As observed in other insects [27], fecundity of non-irradiated *D*. *suzukii* females was similar for those mating with non-irradiated or irradiated males for all doses tested. This implies that wild females mated with irradiated males would still damage the fruits when laying sterile eggs, and these oviposition scars would be an origin for bacterial and fungal infections [5]. However, this drawback will gradually wane along with the reduction of wild female *D*. *suzukii* populations resulting from the release program. Released irradiated females do not oviposit eggs, however it remains unclear if they still pierce the fruits, for feeding. If they do, the development of a genetic sexing strain that produces only males, like the one for the Mediterranean fruit fly [52], could be an option not only to avoid producing sterile females, but also to reduce production cost and enhance efficiency of sterile males.

The relationship between *D*. *suzukii* male fertility (expressed as egg hatch) and irradiation dose is similar to what has been observed in other insects, i.e. a rapid decrease of fertility with increasing dose rates [53], with the lowest fertility (4.0%) obtained with a dose of 120 Gy. Our study provides the first dose-response curves for *D*. *suzukii*. When examining the use of irradiation for quarantine purposes on fresh fruits infested with *D*. *suzukii*, Follett et al. [20] concluded that a dose of 80 Gy applied to late-stage pupae completely prevented reproduction of emerging adults. In our study, we tested the sterility of *D*. *suzukii* males and females separately, by mating them with non-irradiated individuals of the opposite sex instead of mating irradiated males with irradiated females.

Although very few individuals were available to explore aspects of the fertility of the F1 generation, our results suggest that offspring of partially sterile males and non-irradiated females are fertile. Therefore, selecting an optimal irradiation dose for *D*. *suzukii* must be based on the dose-response of the parental F0 generation, and not on inherited sterility since it was not observed in this species. Most cases of inherited sterility have been observed in lepidopteran species [48], but partial inherited sterility has also been shown in the large milkweed bug *Oncopeltus fasciatus* Dallas (Hemiptera: Lygaeidae) [54]. The pattern of sterilization of *D*. *suzukii* following irradiation is therefore similar to patterns observed in other fruit flies belonging to the *Tephritidae* family.

This study is the first to consider the SIT as a control technique for *D*. *suzukii*. Irradiated sterile males could be released in the environment to reduce *D*. *suzukii* populations in targeted areas. They are expected to mate with wild females and prevent them from producing descendants. Further research is required, for instance to examine competitiveness of irradiated males [29]. Experiments have been undertaken to compare the mating behavior of irradiated and non-irradiated males in the laboratory. The irradiation dose of 120 Gy seems promising as it sterilizes *D*. *suzukii* pupea without reducing their emergence and longevity or increasing the rate of deformed flies.

## Supporting information

S1 Table Supporting data of the adult emergence and deformity experiment. (XLSX)

S2 Table Supporting data of the longevity experiment. (XLSX)

S3 Table Supporting data of the fecundity experiment. (XLSX)

S4 Table Supporting data of the fertility experiment. (XLSX)

S5 Table Supporting data of the F1 survival experiment. (XLSX)

S6 Table Supporting data of the F1 fertility experiment. (XLSX)

S7 Table Supporting data from an unpublished previous test of non-irradiated D. suzukii survival on carrot diet. (XLSX)
